# Supplementary material for: Quantifying the shift of public export finance from fossil fuels to renewable energy
Source: Nat Commun. 2025 Jan 29;16:900. doi: 10.1038/s41467-025-55981-0 (PMC11779918; doi:10.1038/s41467-025-55981-0)
Supplement: Supplementary file 1 — Supplementary Information [file 41467_2025_55981_MOESM1_ESM.pdf]

Supplementary Note 1

The global export finance system and the OECD Arrangement. Following decades of export-led growth, export financing has a significant place in modern economic systems. One should distinguish between public ECAs holding government mandates and private insurers or banks that are operating similarly. Public ECAs can relatively safely compensate exporters or lenders (e.g., in the case of repayment loss) since they can draw up a debt settlement arrangement with the Paris Club, a group of creditors that collects public debt. This, together with the convening power and general political umbrella, makes public (or 'official') ECAs attractive business partners for exporters or foreign buyers. The Berne Union[1], the global association for the export credit and investment insurance industry, currently lists 84 member organizations, of which 40 are listed as official ECAs in the Export Credit Group (ECG)[2], a working group at the OECD. The OECD furthermore provides a forum for the main regulatory body in official export financing, the Arrangement on Officially Supported Export Credits or, in short, the "OECD Arrangement".[3,4] This framework provides common terms and conditions for OECD Arrangement Participants in key strategic sectors, like ships, aircraft or renewable energies, and aims thereby to enable a 'level-playing field'.[3] Within the OECD Arrangement, in 2021, a ban on supporting unabated coal-fired electricity generation was adopted[5] – the first time the framework was used as a policy tool in a prohibitive manner. However, no similar restrictions exist for oil and gas sectors, which remains a significant bottleneck for climate ambitions.[6,7] The Participants in the Arrangement include Australia, Canada, the European Union, Japan, Korea, New Zealand, Norway, Switzerland, Türkiye, the United Kingdom, and the United States. Hence, not all ECG countries are Participants in the Arrangement, and neither are important non-OECD countries like China. Currently, the international cooperation between Participants and important non-Participants, such as China, India, and Indonesia, is suspended.[8]

Supplementary Note 2

Implications for fossil fuel-export dependent countries. We used an approach by the United Nations Development Programme[9] that classifies 40 countries worldwide as 'highly dependent' on fossil fuel exports based on a three-part measure: (1) fossil fuel rents as a percentage of GDP; (2) fossil fuel exports as a percentage of total exports; and finally, (3) fossil fuel-related resource revenues as a percentage of total government revenue. Rents are here understood as the difference between the cost of extraction and the typical (market) price of sale.[10] Between 2015-2019, these countries exhibit a mean fossil fuel rent-to-GDP ratio of 14.3%, a mean value of fuel exports over total exports of 61.2%, and a mean fossil fuel-related resource revenue over total government revenue of 38.6%. The full list of countries and indicators is available in the above cited publication. To understand the extent to which ECAs support projects in fossil fuel-export dependent countries, we calculated the share of cumulative ECA commitments received by the above list between 2013 and 2023. In the following table, we list the 25 highly fossil fuel-export dependent countries that are supported by ECAs (in descending order by fossil commitments):

		**Cumulative ECA commitments in USD2020 billion**		
**#**	**ECA project host country**	**Fossil**	**RET**	**Grid**
1	United Arab Emirates	20.82	0.82	1.96
2	Mozambique	19.66	-	-
3	Russian Federation	18.81	0.29	-
4	Kuwait	11.34	-	-
5	Egypt	10.51	1.10	1.18
6	Saudi Arabia	10.03	0.63	1.25
7	Oman	5.15	0.12	-
8	Nigeria	5.14	1.01	-
9	Bahrain	4.39	-	-
10	Norway	4.31	0.36	-
11	Malaysia	4.03	0.17	-
12	Ghana	2.31	0.01	0.07
13	Angola	2.05	3.61	1.67
14	Uzbekistan	1.79	0.00	-
15	Turkmenistan	1.61	-	-
16	Iraq	1.44	-	2.00
17	Colombia	0.94	0.04	-
18	Azerbaijan	0.56	-	-
19	Mongolia	0.24	0.05	-
20	Kazakhstan	0.23	-	-
21	Qatar	-	0.17	-
22	Bolivia	-	0.13	-
23	Congo, Democratic Republic of the	-	0.09	-
24	Ecuador	-	0.00	-
25	Cameroon	-	-	0.32

Supplementary Table 1

Country and organizational coverage. Overview of all 31 countries and 45 ECAs included in this publication as well as their participant status as of February 2024 in the OECD Arrangement and/or major climate coalitions in export finance, namely the Export Finance for Future (E3F) coalition, the Clean Energy Transition Partnership (CETP), sometimes referred to as the 'Glasgow Statement', and the UN-convened Net Zero Export Credit Alliance (NZECA). We count organizations separately when ECA names or mandates change over time (e.g., France or Norway).

Country (alphabetical order)	ECAs covered	OECD Arrangement Participant	E3F climate club member	CETP signatory	NZECA member
Australia	EFA - Export Finance Australia	X		X	
Austria	OeKB - Oesterreichische Kontrollbank Aktiengesellschaft	X	X		
Belgium	Credendo	X	X	X	
China	Sinosure - China Export & Credit Insurance Corporation<br>Export-Import Bank of China				
Czech Republic	Czech Export Bank<br>EGAP - Export Guarantee and Insurance Corporation	X		X	
Denmark	EIFO - Denmark's Export and Investment Fund<br>ELO - Eksportlaaneordningen	X	X	X	X
Finland	FEC - Finnish Export Credit<br>Finnvera plc	X	X	X	
France	Bpifrance<br>COFACE (until 2017)	X	X	X	
Germany	Euler Hermes Aktiengesellschaft	X	X	X	
Hungary	EXIM Hungary - Hungarian Export-Import Bank	X			
India	ECGC - Export Credit Guarantee Corporation Limited<br>Export - Import Bank of India				
Indonesia	Indonesia Eximbank				
Italy	SACE - Servizi Assicurativi per il Commerico Estero	X	X	X	
Japan	JBIC - Japan Bank for International Cooperation<br>NEXI - Nippon Export Investment Insurance	X			
Korea	KEXIM - Export Import Bank of Korea<br>KSURE - Korea Trade Insurance Corporation	X			
Luxembourg	ODL - Luxembourg Export Credit Agency	X			
Malaysia	MEXIM - Export-Import Bank of Malaysia Berhad				
Mexico	BANCOMEXT - Banco Nacional de Comercio Exterior	X			
Netherlands	ADSB - Atradius Dutch State Business	X	X	X	
Norway	Eksfin - Export Finance Norway<br>Eksportkreditt Norge AS and GIEK (until 2021)	X		X	
Poland	KUKE - Export Credit Insurance<br>Corporation Joint Stock Company	X			
Russian Federation	EXIAR - Export Insurance Agency of Russia<br>Roseximbank				
Saudi Arabia	Saudi EXIM Bank				
South Africa	ECIC - Export Credit Insurance Corporation of South Africa				
Spain	CESCE Credit Insurance	X	X	X	(X)
Sweden	EKN - The Swedish Export Credit Agency<br>SEK - Swedish Export Credit Corporation	X	X	X	X
Switzerland	SERV - Swiss Export Risk Insurance	X		X	
Thailand	THAI EXIMBANK - Export-Import Bank of Thailand				
United Arab Emirates	ADEX - Abu Dhabi Exports Office				(X)
United Kingdom	UKEF - UK Export Finance	X	X	X	X
United States	US EXIM BANK - Export-Import Bank of the United States	X		X	

Supplementary Table 2

Definition of energy sector. Since 2022, ten European countries report energy-related export finance support via the Joint Transparency Reporting.[11] Given its suitable logic, we adopted the E3F approach, with the only notable difference being that we report grid (transmission infrastructure and storage) as well as nuclear and hydrogen projects separately. Note that TXF data only includes few nuclear (n=4) and hydrogen deals (n=4, out of which n=3 are considered 'green' hydrogen).

**Value chain**	**Disaggregation**			
**Fossil fuels**	Coal, oil, gas			
Segment	*Upstream* Exploration, Production	*Midstream* Processing, Storage, Transport	*Downstream* Marketing, Distribution, Refining	*Power generation*
**Renewable energy technologies (RETs)**	Wind, solar, hydro, biomass, biofuels, waste-to-energy, green hydrogen			
Segment	*Power generation*			
**Other energy-related activities**	Nuclear energy, Conventional hydrogen		Grid	
Segment	*Power generation*		*Transmission, distribution, and storage*	

Supplementary Table 3

Overview of alternative data sources and prior empirical studies. Overview of relevant papers published by non-governmental organizations, governmental/international organizations, or peer-reviewed articles. Notation in the table: X = fully covered; (X) = covered with reservations.

		Major ECA countries or groups covered											Steps of energy value chains covered				Details	
**Publications**	**Years covered**	Japan	South Korea	China	United States	Canada	Italy	Denmark	Germany	Other E3F members	Other non-E3F members	*Total # of countries covered*	Full value chain - fossil	Renewable technologies	Nuclear and hydrogen	Grid	Split by financial instrument	Split by recipient country
Non-governmental organizations																		
Oil Change International[a]	2006–2022	X	X	-	X	X	X	-	X	(X)	(X)	16	X	X	-	X	(X)	(X)
Perspectives Climate Research[b]	Varying	X	X	(X)	X	X	X	-	X	(X)	(X)	11	-	-	-	-	(X)	-
Both ENDS[c]	Varying	-	-	-	-	-	-	-	-	X	-	1	-	-	-	-	-	-
Natural Resource Defence Council[d]	2007-2015	-	-	-	-	-	-	-	-	-	-	-	-	-	-	-	-	(X)
Governmental/international organizations																		
OECD Export Finance Reporting[e]	1999-2022	X	X	-	-	X	X	X	X	X	(X)	39	-	X	X	-	-	-
E3F Joint Transparency Reporting[f]	2015–2022	-	-	-	-	-	X	X	X	X	-	10	X	X	X	X	-	-
Berne Union[g]	2019-2023	(X)	(X)	(X)	(X)	(X)	(X)	(X)	(X)	(X)	(X)	?	-	-	-	-	X	X
Peer-reviewed																		
Liao 2021	2013-2018	*Uses data from the Natural Resource Defence Council.*										-	-	-	-	-	-	-
Klasen et al. 2022	2021-2030	*Extrapolate data from surveys and the Climate Policy Initiative.*										-	-	-	-	-	-	-
Peterson and Downie 2023	2006-2020	*Use data from Oil Change International.*										-	-	-	-	-	-	-
This article[h]	2013–2023	X	X	X	X	-	X	X	X	X	(X)	31	X	X	(X)	X	X	X

Notes:

[a] Oil Change International maintains the Public Finance for Energy database (https://energyfinance.org/#/data).[6,12,13]

[b] Perspectives Climate Research conducted eleven country case studies (https://perspectives.cc/initiative/eca/).[14–24]

[c] Both ENDS regularly publishes studies on Atradius Dutch State Business, the Dutch ECA.[25]

[d] Use and collect data together with Oil Change International. Focus on coal only.[26]

[e] The OECD reporting involves aggregate cash flows by ECAs from 25 EU members (incl. UK) and 14 non-EU OECD countries in Special Drawing Rights. The reporting covers short-term credits as well as medium- and long-term (no energy-/ sector-specific reporting).[27]

[f] Part of the European climate club "Export Finance for Future (E3F)".[11,28]

[g] The Berne Union is the umbrella association for the global export credit and investment insurance industry. No country- or energy-specific reporting.[29–31]

[h] Note that the number of countries in this article (31) refers to countries with public ECAs active in the energy sector. Besides Canada, which does not report to TXF, we excluded countries and ECAs not active in the energy sector.

Supplementary Table 4

Literature review. We conducted a literature review in Web of Science using the search terms 'Export finance' OR 'Export credit' with 'Energy' OR 'Climate' OR 'Green' in titles (n=22 results) and abstracts (n=380 results). We screened the titles and abstracts of all papers and identified twelve articles on ECAs with direct relevance to the energy-climate nexus. We added a paper not captured by this query that we deem nonetheless directly relevant (Manych et al., 2023). In the table below, we present the gist of these articles as well as the complementarity of our contribution in descending chronological order.

**Article**	**Type of article**	**Data used**	**Most relevant findings**	**Complementarity of the present contribution**
Klasen et al. (2024)[32]: Navigating geopolitical and trade megatrends: Public export finance in a world of change	Empirical and conceptual	Interviews, official documents and policy analysis	ECAs are adapting to rising geopolitical tensions, globalization, and climate change by shifting from traditional roles to proactive trade facilitators.<br><br>ECAs increasingly align with national industrial policies, particularly in strategic sectors like climate-related technologies.<br><br>Introduction of new financial products (e.g., untied loans, working capital guarantees) to meet evolving exporter needs.	This paper discusses broader shifts in the role of ECAs, which our paper supports with empirical data and a specific focus on the energy sector.
Peterson and Downie (2024)[33]: The international political economy of export credit agencies and the energy transition	Commentary (academic)	Case studies of lending by the United Kingdom Export Finance Department (UKEF) for fossil fuel and renewable energy projects as well as aggregate data from Oil Change International.	ECAs have historically supported carbon-intensive sectors, but they also hold the potential to drive the clean energy transition.<br><br>ECAs have been largely overlooked in the literature of International Political Economy (IPE), despite their substantial influence on global energy finance.<br><br>Research should focus on how global and national climate governance efforts incorporate or fail to incorporate ECAs, and the effectiveness of such efforts.	This commentary highlights export finance in the energy sector as a major research gap in IPE scholarship, which we fill empirically with the present contribution.
Klasen and Vassard (2023)[3]: The new OECD arrangement on export credits: Breakthrough or bad compromise?	Commentary (practitioner)	Literature review and policy analysis	This analysis highlights the mixed outcomes of the new OECD Arrangement on export credits, recognizing it as a significant step forward while also pointing out areas where further reform and clarity are needed	Our paper provides nuanced evidence of where the "green" shift within export finance in OECD countries and beyond is occurring and how.
Klasen et al. (2022)[34]: Export finance and the green transition	Empirical	Surveys from 20 EXIMs and ECAs<br><br>Secondary data from publicly available sources such as Climate Policy Initiative (CPI)	EXIMs and ECAs strongly support climate-action-related transactions with financing, guarantees, and insurance amounting to EUR 6.7–8.4 billion in 2020, surpassing previous CPI estimates.<br><br>EXIMs and ECAs need to increase their climate financing 6.8 times, reaching EUR 45.3–57.4 billion by	This paper provides context to our paper regarding the importance of ECAs for the energy transition and the need to scale up climate finance. Our paper provides the comprehensive empirical data documenting where ECAs stand with regard to climate finance contributions.

		EXIM and ECA annual reports for the period 2018–2020	2030, to meet the climate finance volumes required.	
Jansen (2022)[35]: The role of the OECD export credit arrangement	Empirical and conceptual	Aggregate historical data on export credits supported under the OECD Arrangement from 2007–2021 (OECD database)<br>Reports and studies on the financing activities of the Chinese Development Bank and Chinese Export–Import Bank<br>Analysis of recent policy developments and negotiations related to the green energy transition	The OECD Arrangement on Officially Supported Export Credits plays a critical role in maintaining trade flows and supporting international trade, especially during times of crisis.<br>The Arrangement has supported USD 717 billion in export credits from 2007 to 2021, with energy-related projects being a significant portion.<br>There has been a shift from supporting fossil fuel projects to renewable energy projects, with renewable energy support increasing significantly since 2018.<br>In 2020 and 2021, support for renewable energy projects was more than five times that of nonrenewable projects.	While this paper provides some high-level aggregate data on export finance in the energy sector based on the OECD database, our paper complements it with a more granular and previously unpublished ECA finance data disaggregated by country, energy type, steps of the value chain, financial instrument, etc.
Lundquist (2022)[36]: Export credit agencies delivering finance for the green transition in times of crisis.	Empirical and conceptual	Case studies and experiences of EKF (Denmark's Export Credit Agency)<br>Statistics on EKF's financing of renewable energy projects, especially wind energy<br>Policy and regulatory framework analysis	ECAs like EKF play a crucial role in financing the green transition by providing risk capital and long-term funding.<br>ECAs need to continuously adapt their business models, products, and competencies to meet the evolving demands of the green transition.<br>Continuous updating and recalibration of government policies and regulatory frameworks are necessary to incentivize green solutions and support the green transition.	This paper provides context to our paper regarding the importance of ECAs for the energy transition and the need for international collaboration to align ECA policies with the energy transition needs.
Manych et al. (2023)[37]: Pushed to finance? Assessing technology export as a motivator for coal finance abroad	Empirical and conceptual	Global Coal Plant Tracker: For data on coal units.<br>World Electric Power Plants Data Base<br>Global Coal Project Finance Tracker<br>Semi-structured interviews	Both public and private financial institutions continued to fund coal plants even after the Paris Agreement, although there is a noted downward trend in overall financial commitments.<br>Primary Financing Countries: China, Japan, and South Korea are the dominant sources of cross-border debt financing for coal plants, particularly through public banks.<br>Although countries like China, South Korea, and Japan have pledged to cease financing coal plants abroad, the implementation and adherence to these commitments remain uncertain, especially in the context of recent global energy market disruptions due to the Russian invasion of Ukraine.	Our paper expands this analysis by providing more granular data on coal, oil, and gas export finance.
Michie (2022)[38]: The role of the global financial system in financing the	Commentary (practitioner)	Analysis of policy documents and case studies	While some ECAs have committed to net-zero targets by 2050, broader participation is needed. ECAs can learn from private sector initiatives	The commentary discusses the policy reforms necessary to align ECAs with the Paris Agreement. Our paper

transition to net zero			like GFANZ to develop and implement effective net-zero strategies.<br><br>ECAs should create comprehensive net-zero transition plans, similar to the private sector's adoption of climate-related financial disclosures. This would involve setting clear decarbonization strategies and targets.	provides empirical evidence to support it.
Hopewell (2021)[39]: Negotiating in the Dragon's Shadow: Export Credit for Coal Plants	Conceptual (book chapter)	Policy analysis	The absence of China, a major player in export credit for coal power plants, significantly undermines efforts to create effective global trade rules. This reflects broader difficulties in global governance when major economies are not part of key agreements.<br><br>The reluctance of OECD countries to agree to restrictive measures without China's involvement limited the scope and ambition of the resulting agreement.	This book chapter highlights the importance of China's involvement in international policy processes around ECAs, for which our article provides empirical justification.
Liao (2021)[40]: The Club-based Climate Regime and OECD Negotiations on Restricting Coal-fired Power Export Finance	Empirical and conceptual	Primary data from interviews with people involved in the negotiations<br><br>Secondary data from various sources including OECD reports and documents	The US led the negotiations to restrict export finance for coal-fired power projects within the OECD.<br><br>Japan and South Korea resisted the US's push for stricter rules due to competitive disadvantages against Chinese ECAs not bound by OECD regulations.<br><br>A 'club-based' approach to climate governance proved ineffective without the inclusion of powerful non-OECD countries like China.	This paper contextualizes the complexity of global climate governance and the need for inclusive and cooperative approaches that engage all major players, particularly China. Our paper builds on it by providing a quantitative analysis of the "green shift" in major OECD and non-OECD countries.
Hopewell (2019)[41]: How Rising Powers Create Governance Gaps: The Case of Export Credit and the Environment	Empirical	Interviews, policy analysis, case study	The rise of non-OECD countries as major exporters and financiers highlights the limitations of the current global governance framework, which was designed primarily for OECD countries.<br><br>There is a pressing need to expand and adapt global governance mechanisms to include emerging powers and ensure that they adhere to environmental standards in their export credit activities.	This paper contextualizes the complexity of global climate governance and the need for inclusive and cooperative approaches that engage all major players, including non-OECD. Our paper builds on it by providing a quantitative analysis of the "green shift" in major OECD and non-OECD countries.
Wright (2011)[42]: Export Credit Agencies and Global Energy: Promoting National Exports in a Changing World	Conceptual	Aggregate OECD data on export credit agencies (ECAs) and their financing activities in the energy sector.<br><br>Analysis of ECA policies.<br><br>Case studies and examples of ECA-supported projects in various countries.	ECAs significantly impact energy policy goals, including expanding energy supply in developing countries and influencing the carbon intensity of energy development.<br><br>The growing influence of ECAs from non-OECD countries challenges the effectiveness and legitimacy of OECD governance arrangements.<br><br>Tensions exist between the national economic objectives of ECAs and their global environmental and social impacts.	This paper provides context to our paper regarding the importance of ECAs for the energy transition and discusses the limitations of OECD-based governance.

Supplementary Table 5

Overview of financial organizations with ECA-like capabilities that were excluded from this analysis. Here, we list all organizations initially included in TXF transaction data but omitted to retain a clean sample scope of ECAs with an official public mandate. We provide a rationale for excluding each organization individually. Organizations listed here can provide a relevant second-order sample for future research.

Name of Organization	Rationale to exclude from public ECA sample
ABGF - Brazilian Export Credit Insurance Agency	*Not within energy scope*
ASHRA - The Israel Foreign Trade Risks Insurance Corporation	*Not within energy scope*
Abu Dhabi Exports Office (ADEX)	*Not within energy scope*
Afreximbank	*Multilateral or other development finance*
Africa Finance Corporation (AFC)	*Multilateral or other development finance*
African Trade & Investment Development Insurance (ATIDI)	*Multilateral or other development finance*
Asian Development Bank (ADB)	*Multilateral or other development finance*
Asian Infrastructure Investment Bank	*Multilateral or other development finance*
BNDES - Brazilian Development Bank	*Bilateral or other development finance*
Bank Gospodarstwa Krajowego (BGK)	*Bilateral or other development finance*
British International Investment (BII)	*Bilateral or other development finance*
Central American Bank For Economic Integration (CABEI)	*Multilateral or other development finance*
China Development Bank	*Bilateral or other development finance*
Corporate Internationalisation Fund (FIEM)	*Multilateral or other development finance*
Deutsche Investitions- und Entwicklungsgesellschaft (DEG)	*Bilateral or other development finance*
Development Bank of Japan (DBJ)	*Bilateral or other development finance*
Development Bank of Southern Africa (DBSA)	*Multilateral or other development finance*
EXIM R - Eximbank of Romania	*Not within energy scope*
Economic Development Cooperation Fund	*Bilateral or other development finance*
European Bank for Reconstruction and Development (EBRD)	*Multilateral or other development finance*
European Investment Bank (EIB)	*Multilateral or other development finance*
FMO Development Bank	*Bilateral or other development finance*
Government of Brazil- Export Finance Program (PROEX)	*Not within energy scope*
HBOR - Croatian Bank for Reconstruction and Development	*Bilateral or other development finance*
ICIEC - The Islamic Corporation for the Insurance of Investment and Export Credit	*Not within energy scope*
IFC Managed Co-Lending Portfolio Programme	*Multilateral or other development finance*

Industrial Development Bank of Turkey (TSKB)	*Bilateral or other development finance*
International Finance Corporation (IFC)	*Multilateral or other development finance*
KfW - Kreditanstalt für Wiederaufbau	*Bilateral or other development finance*
Korea Development Bank (KDB)	*Bilateral or other development finance*
MIGA - Multilateral Investment Guarantee Agency	*Multilateral or other development finance*
Nordic Investment Bank (NIB)	*Multilateral or other development finance*
OPEC Fund for International Development	*Multilateral or other development finance*
PRI Pensionsgaranti	*Private insurance or export finance*
Slovenian Export and Development Bank (SID)	*Not within energy scope*
Shanghai Pudong Development Bank	*Bilateral or other development finance*
TKYB (Turkiye Kalkınma ve Yatırım Bank)	*Bilateral or other development finance*
Turk Eximbank	*Not within energy scope*
U.S. International Development Finance Corporation (DFC)	*Bilateral or other development finance*
UFK (Untied Credit Guarantees Germany)	*Not within energy scope / incomplete reporting*
EDC (Export Development Canada)	*Incomplete reporting to TXF*

Supplementary Table 6

Within-country level analysis of non-OECD countries. This table uses 'filled-up' data for commitment volumes: For each country, we use the highest volume from either OCI or TXF in USD_{2020}. We display the individual within-group country share (non-OECD countries only). Observation period: 2013-2022.

ECA country	Total fossil commitments	Total RET commitments	Total grid commitments	Total nuclear commitments	Sum (total energy commitments)	Within-group share*
China	65,41	40,31	11,53	13,37	130,62	84%
Russian Federation	8,29	0,49		0,12	8,89	6%
India	3,31	1,99	2,61	0,74	8,66	6%
South Africa	2,72	0,10	0,01		2,82	2%
Indonesia	0,96		0,08		1,04	1%
Thailand	0,68	0,35			1,03	1%
Malaysia	0,78				0,78	1%
Saudi Arabia	0,65	0,02			0,67	0%
United Arab Emirates	0,11				0,11	0%

*Note: * non-OECD country ECAs only.*

Supplementary Table 7

Within-country level analysis of OECD countries. This table uses 'filled-up' data for commitment volumes: For each country, we use the highest volume from either OCI or TXF in USD_{2020}. We display the individual within-group country share (OECD countries only). Observation period: 2013-2022.

ECA country	Total fossil commitments	Total RET commit-ments	Total grid commitments	Total nuclear commitments	Sum	Within-group share*
Korea	145,63	4,57	4,30	4,03	158,54	26%
Japan	113,57	22,03	5,17	0,09	140,85	23%
Canada	100,58	7,64	5,26	0,05	113,52	18%
Italy	33,02	5,01	3,78	0,64	42,45	7%
Germany	19,55	12,05	3,81		35,41	6%

United States	27,66	1,78	0,20	0,02	29,67	5%
Denmark	0,56	22,06	1,40		24,02	4%
United Kingdom	13,15	2,37	1,73	0,00	17,25	3%
France	5,06	6,85	1,87		13,79	2%
Norway	9,66	2,28			11,94	2%
Spain	6,22	3,36	0,13		9,70	2%
Netherlands	4,32	1,35			5,66	1%
Sweden	0,37	3,44	1,34		5,15	1%
Mexico	1,37	1,87	0,04		3,27	1%
Switzerland	2,34	0,69			3,03	0%
Australia	0,88	0,36	0,00	0,00	1,25	0%
Finland	0,49	0,04	0,50		1,03	0%
Belgium	0,27	0,43			0,69	0%
Austria	0,04	0,65			0,69	0%
Hungary	0,34				0,34	0%
Czech Republic	0,11	0,19			0,30	0%
Poland	0,10	0,02			0,12	0%
Luxembourg		0,09			0,09	0%
Turkey	0,06	0,01			0,07	0%

*Note: *OECD country ECAs only.*

Supplementary Methods

Methods used for the data triangulation with Oil Change International's (OCI's) Public Finance for Energy database[12] (latest version dated 25th July, 2024) used in Supplementary Figures 1-7. Here, we compare our results with the only other comprehensive (and publicly available) database covering ECA energy finance provided by OCI, a non-governmental organization. This note describes the method used to fill up under-reported volumes that we report in the figures below. We report Export Development Canada, the Canadian ECA, separately in Supplementary Fig. 7. Source data[45] of all Supplementary Figures are publicly available from https://doi.org/10.5281/zenodo.14261240

OCI data scope:

- Observation period: calendar years 2013-2023. We only include deals with financial closure in these calendar years, noting that there are only very few (n=8) deals for 2023.
- Financial instruments: OCI reports financial instruments with the variable 'mechanism' under which some instruments cannot unambiguously be attributed to either guarantees or direct lending. Hence, we establish a third category 'Other instruments' and report the latter

separately (see Supplementary Fig. 3). Note that 98% of commitments in the section 'Other instruments' originates from n=27 deals with total commitments to the fossil fuel sector of USD_{2020} 100 billion by three ECAs, Export Development Canada, K-SURE and KEXIM (Korea).

- Organizations: ECAs only (n=20). We removed two development finance institutions (BNDES and JICA) that were categorized as "Export finance" but that are no official ECAs.
- ECA countries: The ECA countries covered are Australia, Canada, China, France, Germany, Italy, India, Indonesia, South Korea, Mexico, Russian Federation, South Africa, Turkey, United Kingdom, United States, and Japan (n=16).
- Sectors: We only retain sectors congruent with the E3F energy sector definition (see Supplementary Table 2). This meant we remove the following sectors:
    - "Electric Vehicles" (12 observations)
    - "Climate" (1 observation)
    - "Petrochemical" (62 observations)
- Inflation adjustment: OCI reports financial volumes in current USD. For comparability between years, we converted all nominal values to USD_{2020} using the United States Consumer Price Index published by the International Monetary Fund.[43,44]

Method to fill up missing TXF volumes:

OCI does not report unique identifiers that could be matched with TXF data at the deal- or tranche level. For this reason, it is impossible to join the two databases using unique identifiers to avoid double-counting of commitment volumes. To nonetheless combine the two databases, we collapse ECA commitment volumes in both data sets to a given level (e.g., all direct lending commitments by the German ECA to wind energy deals that closed in 2013) and then retain the respectively larger commitment volume between the TXF and the OCI data set. This procedure allows for the retention of commitment volumes where the other source does not report volumes or reports smaller volumes while mitigating the risk of double counting volumes. However, when choosing the level of granularity at which the data is collapsed before retaining the larger volume, there is a trade-off. On the one hand, a more granular level to compare and combine the two data sources should increase precision. On the other hand, it makes the approach more susceptible to double counting if TXF and OCI both cover a deal but classify it slightly differently (e.g., classifying a deal that finances both solar and wind energy as solar in one data set but as wind in the other). Therefore, the resulting volumes are likely overestimated at least for some countries and technologies and may be further distorted by measurement inconsistencies between TXF and OCI, which is why we use the combined data only as a robustness check.

We use three different approaches by collapsing volumes for each pairing of (i) technology (e.g., wind energy), (ii) closing year, (iii) ECA country and (iv) one of the following:

- instrument (direct lending, guarantee, or other instruments) for Supplementary Fig. 3,
- value chain stage for Supplementary Fig. 5, or,

- recipient country for Supplementary Fig. 6.

Mathematically, this procedure can be expressed as follows. Let $x_{i,c,t,a,b,v,r}$ denote the financial commitment by the ECA(s) of country c as part of transaction i that closed in year t, financing the value chain stage v of technology a in recipient country r using financial instrument b. Let the sum of all financial commitments by ECA(s) from country c in year t for technology a using financial instrument b be denoted as $\overline{x}_{c,t,a,b}$
Then, we retain larger commitment volumes between OCI and TXF:

$$\overline{x}_{c,t,a,b} = \max\{\overline{x}^{OCI}_{c,t,a,b}, \overline{x}^{TXF}_{c,t,a,b}\}$$

where the superscripts denote the respective data set used to calculate sums. By replacing b with v or r in the expressions for $\overline{x}$ above, one obtains the mathematical expressions for collapsing at technology-closing year-ECA country-value chain stage levels or technology-closing year-ECA country-recipient country levels instead.

Given missing deal- and tranche-level information in OCI data, we cannot replicate **Fig. 4**.

Supplementary Figure 1

Aggregate trends. Comparison of all commitments in USD2020 by sector (fossil, RETs, grid) between (**a**) OCI data and (**b**) our sample based on TXF data. Observation period: 2013-2023. Note that 2023 is an incomplete reporting year for OCI. For better comparability, in this figure we exclude Export Development Canada, the Canadian ECA, and report its commitments separately below (Supplementary Fig. 7).

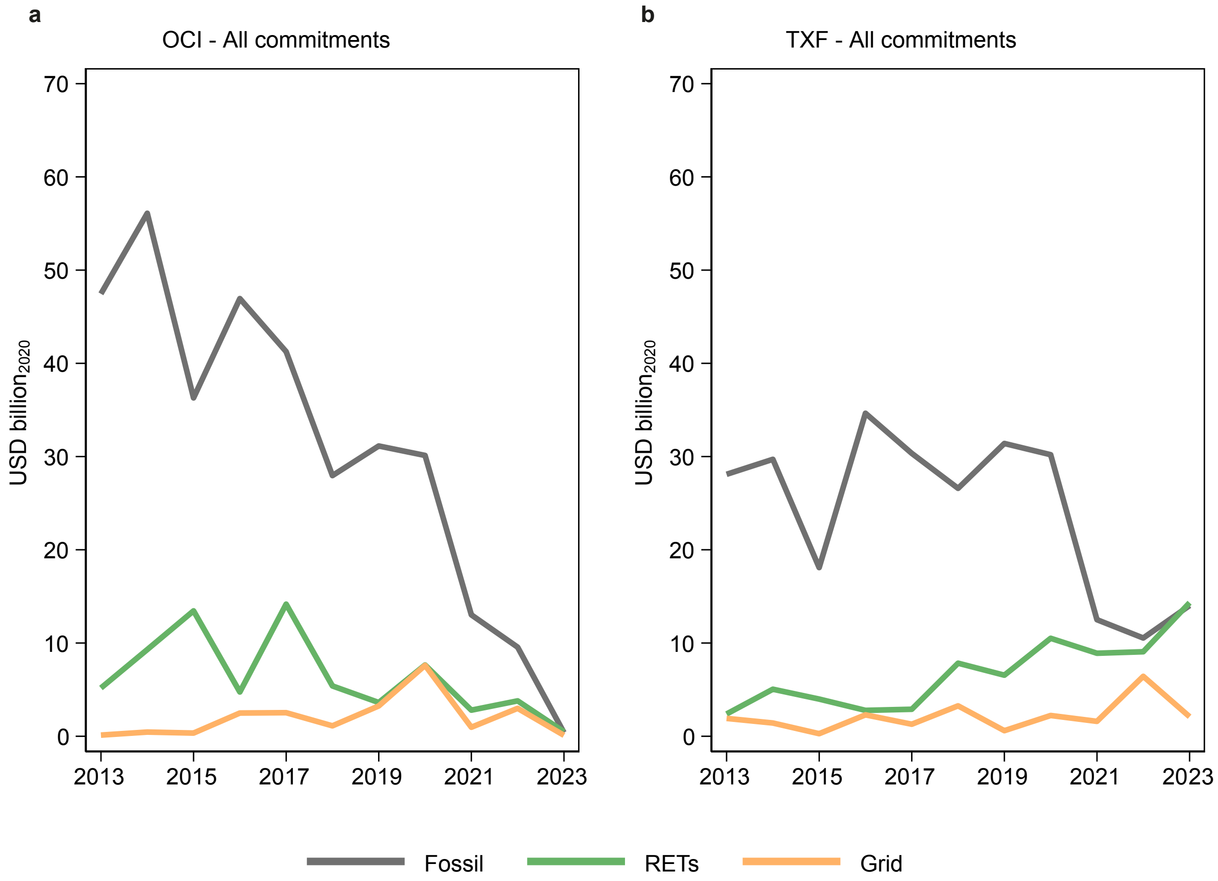

Supplementary Figure 2a

Data triangulation between Oil Change International's Public Finance for Energy database[12] and TXF by country for guarantees. This chart only includes countries whose ECAs have non-zero guarantee involvement in at least one of the two databases. Number of countries shown in this graph: n = 28.

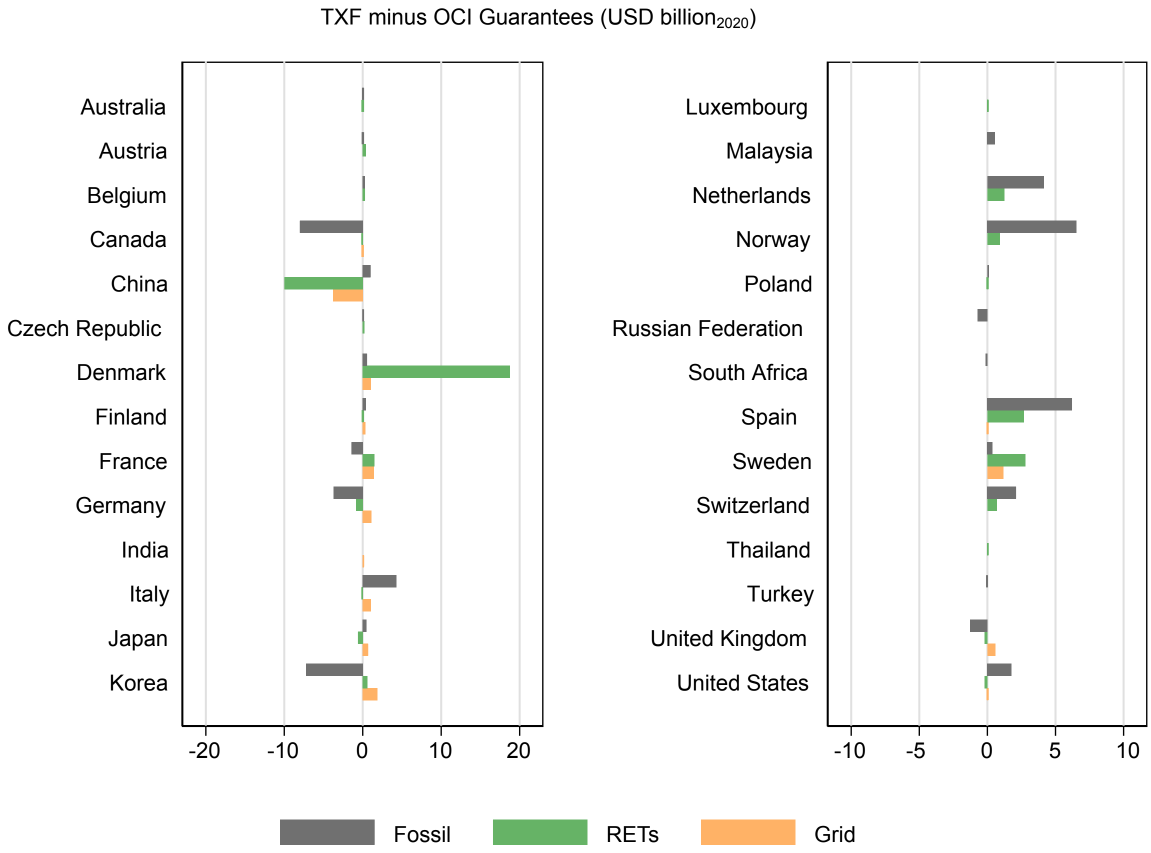

Note: *Given the variability of differences between countries, the scale of X-axis labels varies. Positive values indicate higher coverage for TXF, while negative values indicate higher coverage for OCI. All figures represent cumulative ECA commitments at the country-level between 2013 and 2022.*

Supplementary Figure 2b

Data triangulation between Oil Change International's Public Finance for Energy database[12] and TXF by country for direct lending. This chart only includes countries whose ECAs have non-zero lending involvement in at least one of the two databases. Number of countries shown in this graph: n = 25.

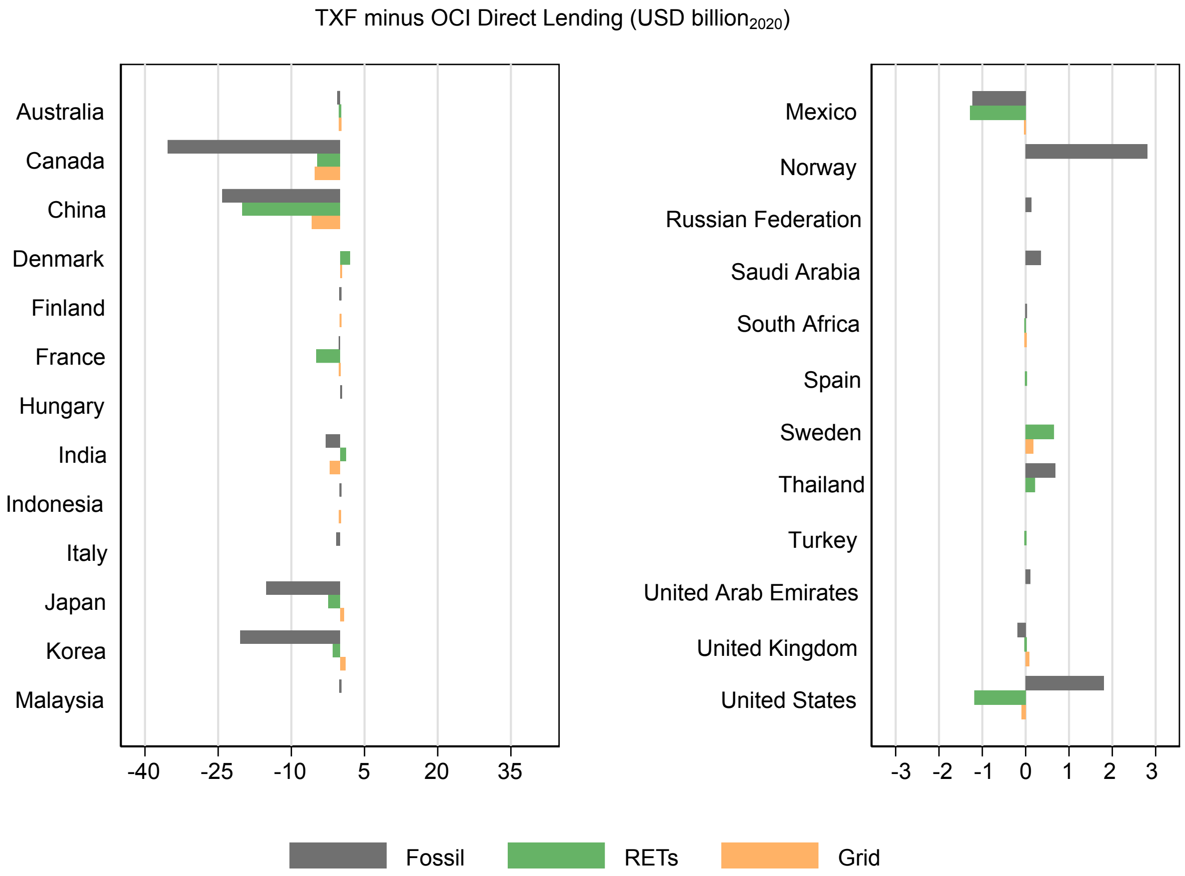

Note: Given the variability of differences between countries, the scale of X-axis labels varies. Positive values indicate higher coverage for TXF, while negative values indicate higher coverage for OCI. All figures represent cumulative ECA commitments at the country-level between 2013 and 2022.

Supplementary Figure 3

(Main Manuscript Figure 1)

Aggregate trends with imputed values from Oil Change International's Public Finance for Energy database[12]. (a) Global sum of ECA energy guarantees by year using imputed values. (**b**) Global sum of ECA direct lending to energy projects by year using imputed values. Area colors indicate different energy subsectors across fossil fuel, renewables, nuclear and grid projects. (**c**) Global sum of 'Other instruments' (shows mainly commitments by South Korean ECAs that could not be further classified). (**d**) Share of RETs over total ECA energy lending and guarantees, respectively. Data coverage is global except Export Development Canada, the Canadian ECA (see Supplementary Fig. 7 below). For 2023 data from TXF, please refer to the Main manuscript and Source Data. Period P1 refers to Pre-Paris (2013-2015), P2 to post-Paris (2016-2019), P3 to the Pandemic (2020-2021), and P4 to post-Glasgow (2022 only). Due to small sample size, we do not display the RET share of 'Other instruments' in Panel D. See Supplementary Source Data.

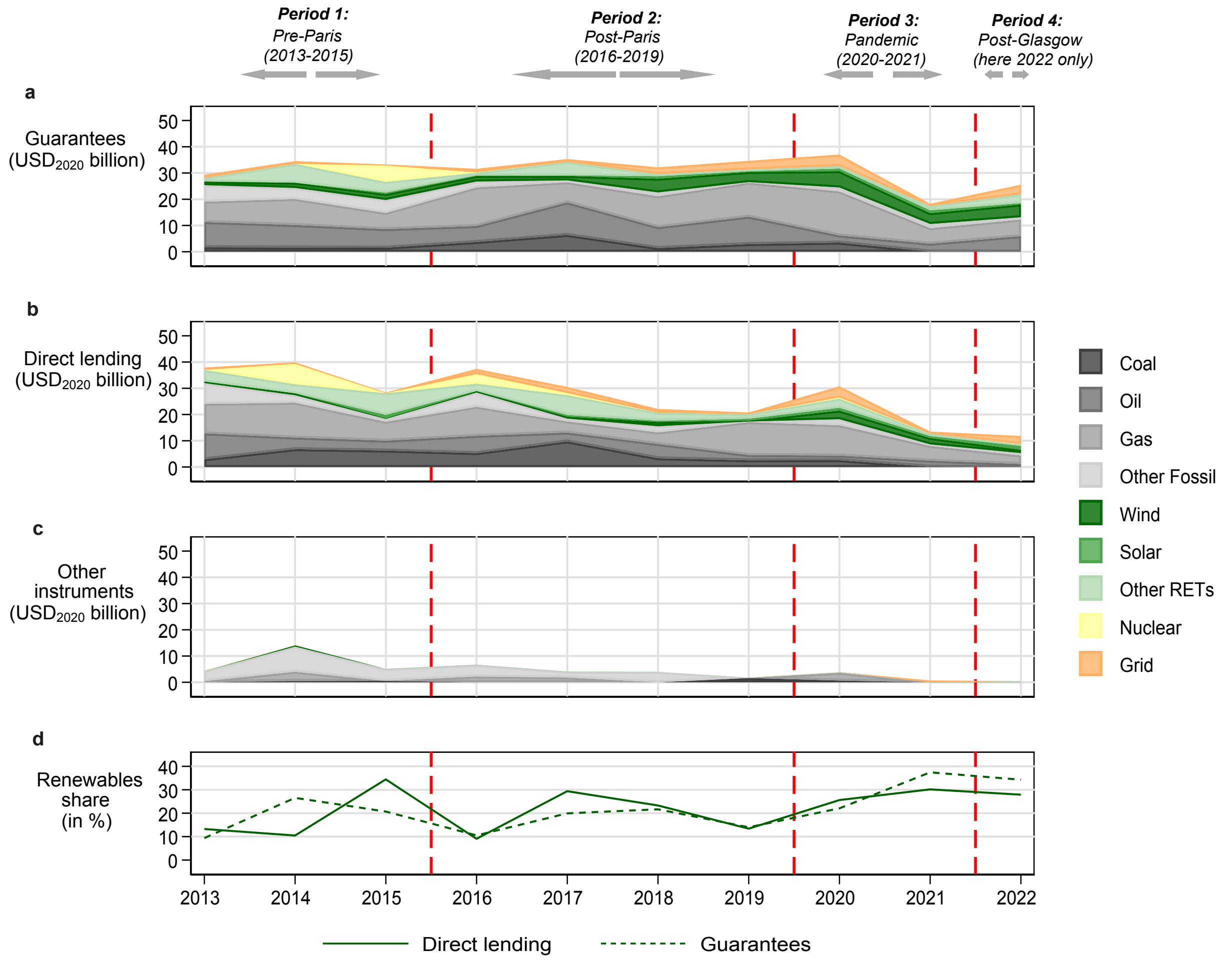

Supplementary Figure 4

(Main Manuscript Figure 2)

Data triangulation between Oil Change International's Public Finance for Energy database[12] **and TXF by value chain stage.** (**a**) Average annual commitments in fossil fuel value chains using imputed values. (**b**) Average annual commitments for renewable power generation projects using imputed values. Commitments aggregate both direct lending and guarantees and are calculated within periods. For comparability, we retain the year 2022 in P4 only. Note that 2023 is an incomplete reporting year for OCI. For better comparability, in this figure we exclude Export Development Canada, the Canadian ECA, and report its commitments separately below (Supplementary Fig. 7). Period P1 refers to Pre-Paris (2013-2015), P2 to post-Paris (2016-2019), P3 to the Pandemic (2020-2021), and P4 to post-Glasgow (2022 only).

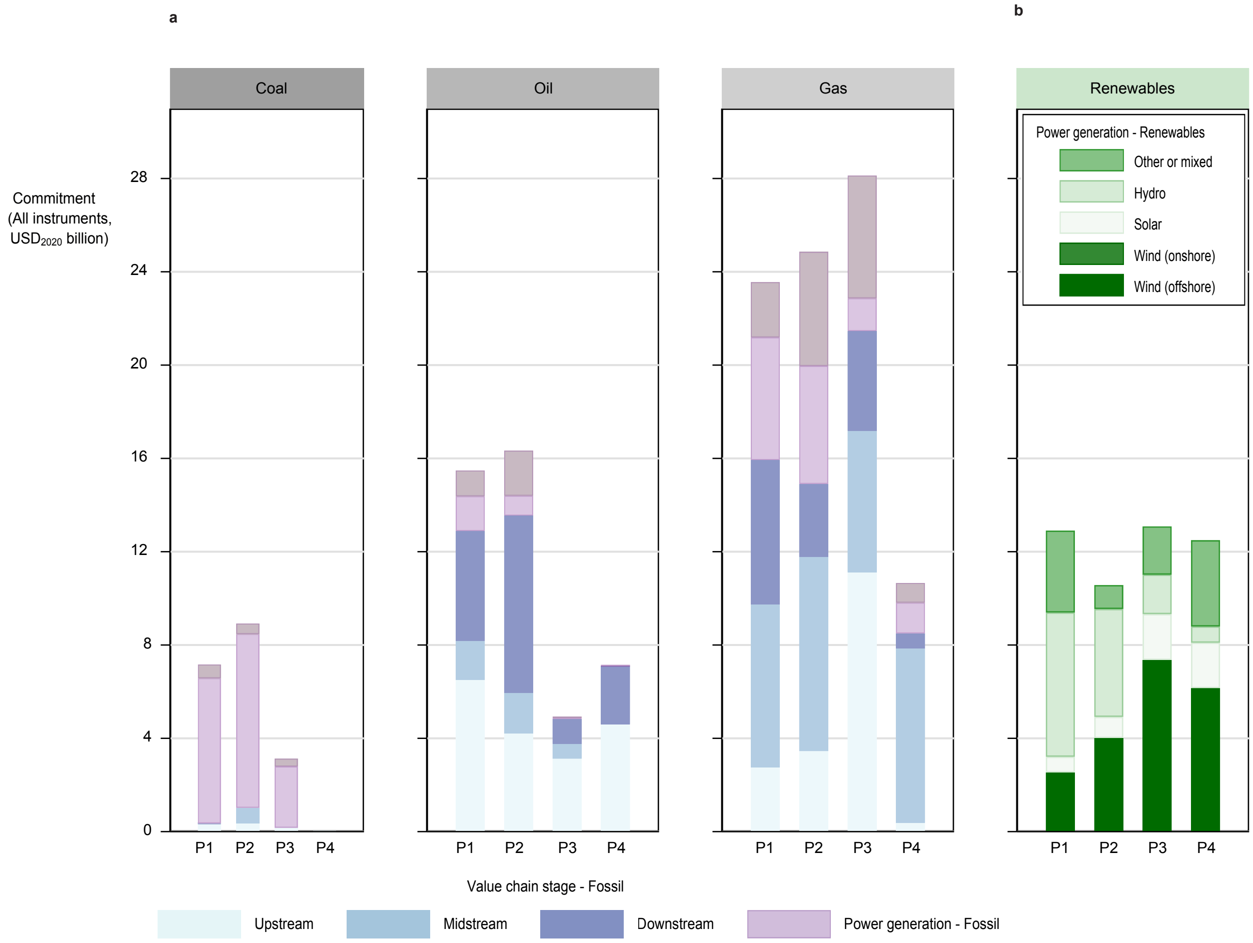

Supplementary Figure 5

(Main Manuscript Figure 3)

Data triangulation between Oil Change International's Public Finance for Energy database[12] **and TXF by country and climate club membership.** (**a**) Comparison of average annual energy commitments between non-E3F versus E3F country groups, by period, using imputed values. (**b**) Share of commitments for fossil fuel projects, RETs, and grid-related projects (incl. storage) in non-E3F countries, using imputed values. (**c**) Share of commitments for fossil fuel projects, RETs, and grid-related projects (incl. storage) in countries of the E3F climate coalition, using imputed values. For comparability, we retain the year 2022 in P4 only. Note that 2023 is an incomplete reporting year for OCI. For better comparability, in this figure we exclude Export Development Canada, the Canadian ECA, and report its commitments separately below (Supplementary Fig. 7). Period P1 refers to Pre-Paris (2013-2015), P2 to post-Paris (2016-2019), P3 to the Pandemic (2020-2021), and P4 to post-Glasgow (2022 only).

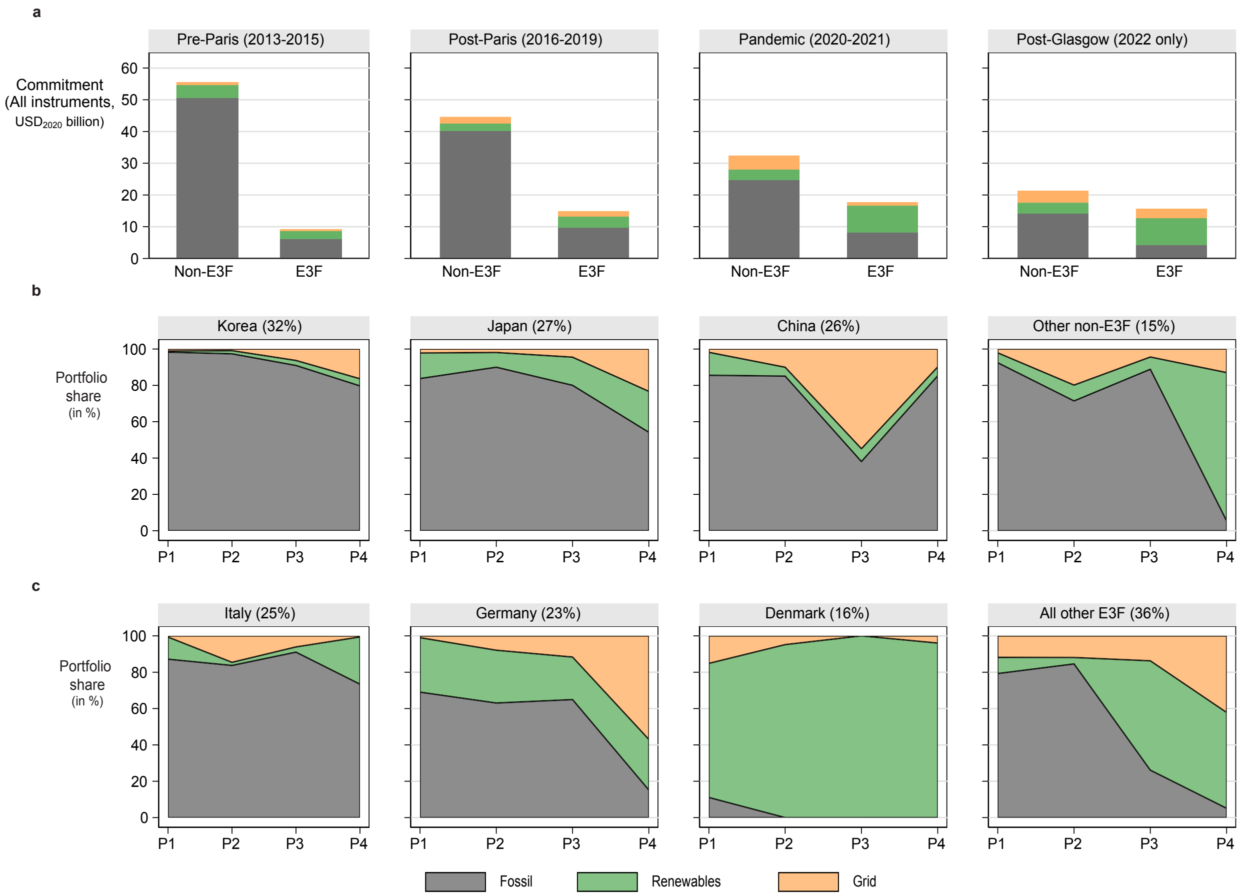

Supplementary Figure 6

(Main Manuscript Figure 5)

Data triangulation between Oil Change International's Public Finance for Energy database[12] **and TXF.** (**a**) Share of each recipient country in the overall 2013-2022 ECA energy finance commitments, including both direct lending and guarantees for fossil fuel and RE projects (using imputed values). Shares are binned for the sake of readability. (**b**) Overall ECA commitments by ECA country over 2013-2022 (using imputed values). Bars indicate the share of commitments received by countries in the same country (red), same region (green), or a different region (blue). Region definitions are taken from TXF and distinguish between Africa, Asia, Asia Pacific, Australasia, Europe, Latin America, the Middle East, North America, and Russia CIS. (**c**) Annual share of recipient countries grouped by the World Bank's income level categorization in overall 2013-2022 ECA energy finance commitments (using imputed values). Note that 2023 is an incomplete reporting year for OCI. For better comparability, in this figure we exclude Export Development Canada, the Canadian ECA, and report its commitments separately below (Supplementary Fig. 7). Natural Earth shapefiles are sourced via Andy South's *rnaturalearth* package.[46] Period P1 refers to Pre-Paris (2013-2015), P2 to post-Paris (2016-2019), P3 to the Pandemic (2020-2021), and P4 to post-Glasgow (2022 only).

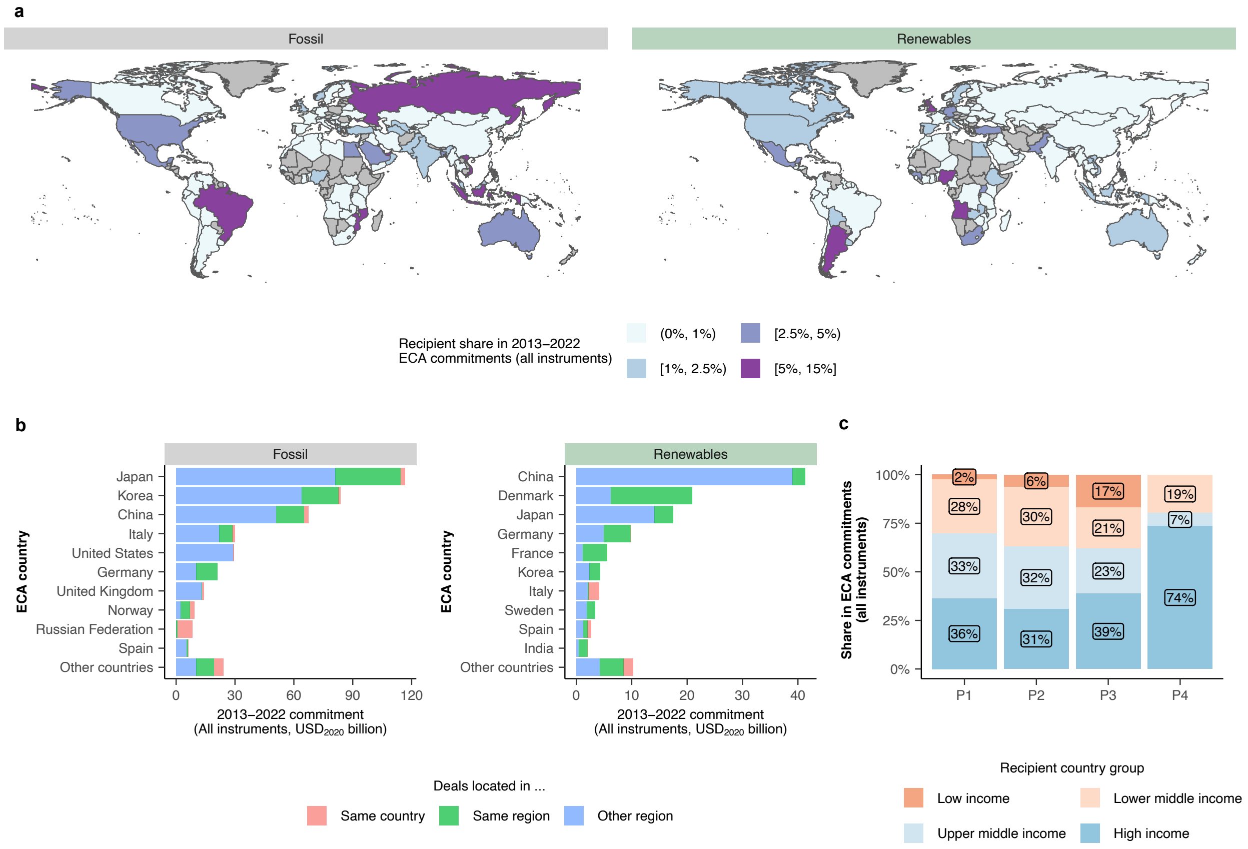

Supplementary Figure 7

Complementary analysis: Canada. Given the incomplete coverage of Export Development Canada in TXF data, we provide an additional analysis on Canada's energy financing portfolio mainly using OCI data. (**a**) Direct energy lending. (**b**) Energy guarantees. (**c**) Other or mixed instruments. (**d**) Portfolio shares of fossil, renewable or grid projects. In imputing the highest value in either OCI or TXF data, we counted n=1 deal from 2014 where TXF reported a 4% higher commitment volume (USD_{2020} 556 million instead of USD_{2020} 533 million), which we include in the data presented below. Period P1 refers to Pre-Paris (2013-2015), P2 to post-Paris (2016-2019), P3 to the Pandemic (2020-2021), and P4 to post-Glasgow (2022 only).

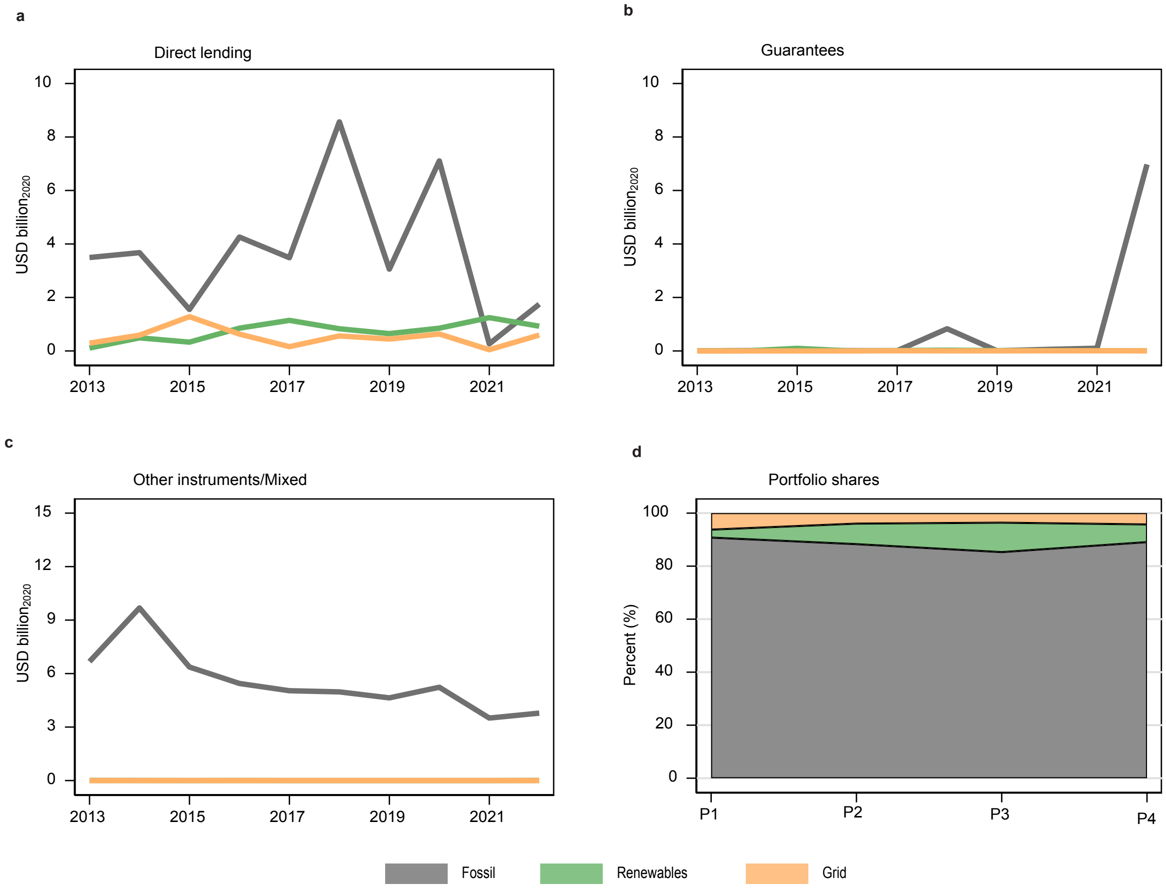

Supplementary References

1. Berne Union. Members. (available at: https://www.berneunion.org/Members) (2024).

2. Organization for Economic Cooperation and Development. Official export credit agencies. (available at: https://www.oecd.org/content/dam/oecd/en/topics/policy-issues/export-credits/official-ecas.pdf) (2023).

3. Klasen, A. & Vassard, J. The new OECD arrangement on export credits: Breakthrough or bad compromise? *Glob. Policy* **14**, 958–961 (2023).

4. Organization for Economic Cooperation and Development. Arrangement on officially supported export credits - version July 2023. (available at: https://one.oecd.org/document/TAD/PG(2023)7/en/pdf) (2023).

5. Organization for Economic Cooperation and Development. Agreement reached at OECD to end export credit support for unabated coal-fired power plants. (available at: https://www.oecd.org/newsroom/agreement-reached-at-oecd-to-end-export-credit-support-for-unabated-coal-fired-power-plants.htm) (2021).

6. Oil Change International. Promise Breakers: Assessing the impact of compliance with the Glasgow Statement commitment to end international public finance for fossil fuels. (available at: https://www.bothends.org/uploaded_files/document/PROMISE-BREAKERS-1.pdf) (2023).

7. Messenger, J. OECD oil and gas export credit ban tabled for next year. *Global Trade Review (GTR)* (available at: https://www.gtreview.com/news/sustainability/oecd-oil-and-gas-export-credit-ban-tabled-for-next-year/) (2023).

8. U.S. Department of the Treasury. Joint statement on the temporary suspension of the technical negotiations in the International Working Group on Export Credits. *Statements & Remarks*. (available at: https://home.treasury.gov/news/press-releases/sm1188) (2020).

9. Jensen, L. Global decarbonization in fossil fuel export-dependent economies - fiscal and economic transition costs. *Dev. Future Ser.* (available at: https://www.undp.org/publications/dfs-global-decarbonization-fossil-fuel-export-dependent-economies) (2023).

10. The World Bank. The changing wealth of Nations 2021: Managing assets for the future. (available at: https://openknowledge.worldbank.org/entities/publication/e1399ed3-ebe2-51fb-b2bc-b18a7f1aaaed) (2021).

11. Export Finance for Future. E3F Status Report 2023. (available at: https://www.ekn.se/globalassets/dokument/hallbarhetsdokument/e3f_status_report_2023.pdf) (2023).

12. Oil Change International. Public Finance for Energy Database. (available at: https://energyfinance.org/#/data) (2024).

13. Oil Change International, O. C. Shift the Subsidies: Financing dirty energy. *Oil Change*

International (available at: http://priceofoil.org/shift-the-subsidies/) (2021).

14. Darouich, L., Censkowsky, P. & Shishlov, I. Paris alignment of export credit agencies: the case of Germany (Euler Hermes) – Perspectives Climate Group. (available at: https://perspectives.cc/publication/paris-alignment-of-export-credit-agencies-the-case-of-germany-euler-hermes/) (2021).

15. Censkowsky, P., Shishlov, I. & Darouich, L. Paris alignment of export credit agencies: The case of the Netherlands (Atradius DSB). (available at: https://perspectives.cc/publication/paris-alignment-of-export-credit-agencies-the-netherlands-atradius-dsb/) (2021).

16. Darouich, L., Censkowsky, P. & Shishlov, I. Paris alignment of export credit agencies: Japan (NEXI and JBIC) – Perspectives Climate Group. (available at: https://perspectives.cc/publication/paris-alignment-of-export-credit-agencies-japan-nexi-and-jbic/) (2021).

17. Censkowsky, P., Shishlov, I. & Darouich, L. Paris alignment of export credit agencies: The case of Canada (Export Development Canada). (available at: https://perspectives.cc/publication/paris-alignment-of-export-credit-agencies-canada-export-development-canada/) (2022).

18. Censkowsky, P., Shishlov, I. & Darouich, L. Paris alignment of export credit agencies: The case of the United States (EXIM). (available at: https://perspectives.cc/publication/paris-alignment-of-export-credit-agencies-usa-exim/) (2022).

19. Brescia, D., Censkowsky, P., Schmidt, M. & Shishlov, I. Paris alignment of export credit agencies: Italy (SACE) – Perspectives Climate Group. (available at: https://perspectives.cc/publication/paris-alignment-of-export-credit-agencies-italy-sace/) (2023).

20. Schmidt, M., Shishlov, I. & Censkowsky, P. Paris alignment of export credit agencies: France (Bpifrance Assurance Export) – Perspectives Climate Group. (available at: https://perspectives.cc/publication/paris-alignment-of-export-credit-agencies-france-bpifrance-assurance-export/) (2023).

21. Darouich, L., Shishlov, I. & Censkowsky, P. Paris alignment of export credit agencies: The case of the South Korea (K-SURE and KEXIM). (available at: https://perspectives.cc/publication/paris-alignment-of-export-credit-agencies-south-korea-the-korea-trade-insurance-corporation-k-sure-and-the-export-import-bank-of-korea/) (2023).

22. Shishlov, I., Censkowsky, P., Shorthouse, R. & Leming, A. Greening UK Export Finance - Bright Blue. (available at: https://www.brightblue.org.uk/wp-content/uploads/2023/03/Greening-UK-Export-Finance.pdf) (2022).

23. Schmidt, M., Censkowsky, P. & Shishlov, I. Paris alignment of export credit agencies: Sweden. (available at: https://pub.norden.org/temanord2024-536/temanord2024-536.pdf) (2024).

24. Schmidt, M., Jia, Z., Weber, L. & Shishlov, I. Paris alignment of export credit agencies:

Finland. (available at: https://norden.diva-portal.org/smash/get/diva2:1888972/FULLTEXT01.pdf) (2024).

25. Both ENDS. Despite violence, 900 million euros in Dutch export support to Van Oord in Mozambique. *Both ENDS*. (available at: https://www.bothends.org/en/Whats-new/Press/Despite-violence-900-million-euros-in-Dutch-export-support-to-Van-Oord-in-Mozambique/) (2021).

26. Chen, H. Carbon Trap: How international coal finance undermines the Paris Agreement. (available at: https://www.nrdc.org/resources/carbon-trap-how-international-coal-finance-undermines-paris-agreement) (2016).

27. Organization for Economic Cooperation and Development. Export credit statistics. *Cash flow results for the period 1999-2022*. (available at: https://www.oecd.org/en/topics/policy-issues/export-credits.html#cashflow) (2024).

28. French Treasury. Seven countries launch international coalition "Export Finance for Future" (E3F) to align export finance with climate objectives. *Direction générale du Trésor*. (available at: https://www.tresor.economie.gouv.fr/Articles/2021/04/14/seven-countries-launch-international-coalition-export-finance-for-future-e3f-to-align-export-finance-with-climate-objectives) (2021).

29. Berne Union. Export credit & investment insurance industry Report 2020. (available at: https://www.berneunion.org/Publication/reports) (2020).

30. Berne Union. State of the Industry report 2021. (available at: https://www.berneunion.org/Publication/reports) (2022).

31. Berne Union. State of the Industry report 2022. (available at: https://www.berneunion.org/Publication/reports) (2023).

32. Klasen, A., Krummaker, S., Beck, J. & Pennington, J. Navigating geopolitical and trade megatrends: Public export finance in a world of change. *Glob. Policy* **15**, 1007-1014 (2024).

33. Peterson, M. & Downie, C. The international political economy of export credit agencies and the energy transition. *Rev. Int. Polit. Econ.* **31**, 978–994 (2024).

34. Klasen, A., Wanjiru, R., Henderson, J. & Phillips, J. Export finance and the green transition. *Glob. Policy* **13**, 710–720 (2022).

35. Jansen, M. Managing the green transition: The role of the OECD export credit arrangement. *Glob. Policy* **13**, 554–556 (2022).

36. Lundquist, P. Export credit agencies delivering finance for the green transition in times of crisis. *Glob. Policy* **13**, 530–533 (2022).

37. Manych, N. *et al.* Pushed to finance? Assessing technology export as a motivator for coal finance abroad. *Environ. Res. Lett.* **18**, 084028 (2023).

38. Michie, A. The role of the global financial system in financing the transition to net zero. *Glob. Policy* **13–4**, 557–562 (2022).

39. Hopewell, K. Power transitions and global trade governance: The impact of a rising China on the export credit regime. *Regul. Gov.* **15**, 634–652 (2021).

40. Liao, J. The club-based climate regime and OECD negotiations on restricting coal-fired power export finance. *Glob. Policy* **12**, 40–50 (2021).

41. Hopewell, K. How rising powers create governance gaps: The case of export credit and the environment. *Glob. Environ. Polit.* **19**, 34–52 (2019).

42. Wright, C. Export credit agencies and global energy: Promoting national exports in a changing world. *Glob. Policy* **2**, 133–143 (2011).

43. Steffen, B. & Schmidt, T. S. A quantitative analysis of 10 multilateral development banks' investment in conventional and renewable power-generation technologies from 2006 to 2015. *Nat. Energy* **4**, 75–82 (2019).

44. International Monetary Fund. Access to macroeconomic and financial data. *Consumer Price Index (CPI)*. (available at: https://data.imf.org/?sk=4ffb52b2-3653-409a-b471-d47b46d904b5) (2024).

45. Censkowsky, P., Waidelich, P., Shishlov, I., & Steffen, B. Scripts and data for "Quantifying the shift of public export finance from fossil fuels to renewable energy" Zenodo. (available at: https://doi.org/10.5281/zenodo.14261240) (2024).

46. South, A. rnaturalearth: World Map Data from Natural Earth. R package version 0.3.4. (available at: https://github.com/ropensci/rnaturalearth) (2023).
